# Surgery of the Vermiform Appendix

**Published:** 1905-02-04

**Authors:** 


### PROGRESS IN MEDICINE AND SURGERY.

### SURGERY OF THE VERMIFORM APPENDIX.

**Appendicitis.**—Macewen [1] affirms that the cæcum and appendix perform an important part in the final stages of digestion. One would therefore expect that serious interference with the function of those parts would give rise to disease. This is exactly what occurs. Though the causes of appendicitis are varied, the majority are intimately associated with the digestive process and the factors controlling it. The differentiation of cæcal indigestion from other forms requires further study before the symptoms pertaining thereto are accurately defined. If from inhibition of the appendicular and cæcal movements, or the want of exudation of the succus entericus, or if the cæcum receives material which the succus entericus cannot digest, a stasis occurs in the contents of the cæcum and the constipation which is so often a feature of the appendicitis ensues. At a later stage fermentative disintegration of the fæcal contents, with absorption of toxins and damage to the wall of the parts is apt to ensue. These are followed by diarrhœa, which is sometimes curative. French [2] considers the limitations of leucocytosis as an indication for laparotomy, and refers to the leucocyte counts in 83 cases of appendicitis. He draws the following conclusions:—The value of leucocytosis in relegating a given case of appendicitis to its proper group, and in deciding whether an operation should be performed or not, is apt to be overrated. Its value, judged from his cases, is even less than that deduced by other recent observers from the figures they have found. Many cases, with 20,000 leucocytes have resolved spontaneously; many, with 15,000 or less, have had pus present. At the same time, leucocyte-counts have afforded valuable evidence in certain cases. In no case where the leucocytes have reached 35,000 has pus been absent. A rising count is of more importance than is the absolute number. Above all, leucocytosis is to be regarded as but one clinical sign amongst many. By itself it may mislead, but taken in conjunction with the pulse-rate, the temperature, and the general condition of the patient, it is an additional sign which may be most valuable in the diagnosis of a difficult case. McCosh [3] says in children the progress of the disease is marked often by its insidiousness. Apparently a mild case will drift almost without signs into a desperate one with general peritonitis; in children there seems to be less tendency for the inflammation to become localised. For these reasons he advises immediate operation in children at any stage of the disease. Cotterill,[4] in addition to operation in fulminating cases, considers that in other less acute cases the following symptoms usually indicate the necessity for immediate operation:—1. Vomiting continuing after the first few hours. 2. Pulse of a hundred or over, quickening, getting weaker, and out of proportion to the temperature. 3. Temperature suddenly falling, or markedly rising after the second day, especially when combined with remissions. 4. Pain suddenly stopping, or after the second day markedly increasing. The general conclusions as viewed from the surgical standpoint appear to be as follows: (1) In fulminating cases, operate at once. (2) In other cases, if seen within the first 24 hours, immediate operation is not only comparatively safe, but probably the safest line of action. (3) In cases seen after 36 hours or more are past, it is probably safest to attempt to wait for a quiet interval; but operation must be immediately performed on the appearance of untoward symptoms. (4) In all cases operate at once if the patient is far out of the way, in the country, etc., and cannot be under the observation of the surgeon. Brook [5] formulates the following conclusions: (1) The very great majority of cases will get over the attack if treated on simple medicinal lines. (2) During the quiet period the appendix should be removed, so as to obviate another attack. (3) During an attack the patient should be carefully and frequently watched, and on any sign of fulmination, operation should at once be done. (4) If there are signs of suppuration, an attempt should be made to tide the patient over the fifth day, and then the pus should be evacuated, if possible, without opening the general peritoneal cavity. If the appendix presents in the wound it should be removed, but no elaborate search should be made for it. It may be removed later on. (5) In general suppurative peritonitis the use of drainage, massive infusions, and calomel. Cumston[6] says the ideal thing which is accomplished occasionally is the removal of the appendix after an attack of appendicular colic, colic without a rise of temperature, and due to the diseased condition of the appendix. Regarding the question of the removal of the appendix during the first twenty-four hours of the first attack, he says he has no hesitancy in saying that it is the ideal method. Stoner[7] claims that the clinical symptoms are of small significance in determining the pathological conditions of appendicitis, and therefore thinks that early operation is desirable. Coe[8] touches upon the following points in a brief *résumé*: Appendicitis is a frequent complication of inflammatory disease of the adnexa. In most cases it is secondary to adnexal trouble, a long appendix adherent to, or in contact with, the right tube or ovary being infected by extension through its wall or through the lymphatics. In a small proportion of cases the infection extends from the appendix to an adherent tube or ovary or cystoma. The symptoms of the associated conditions are usually determined by the more prominent lesion, but they are referable principally to the diseased adnexa. The diagnosis is made from the history, the location of the pain (above, as well as below, the pelvic brim), and the presence of an induration which can be traced from the appendicular region downwards into the pelvis. In acute cases, with an extra pelvic mass, the lateral incision is preferable, with subsequent exploration of the pelvis and vaginal drainage if possible. If the abscess is mainly intra-pelvic and easily accessible, vaginal section is indicated. In subacute and recurrent cases a median incision is to be elected. Bunts[9] records three cases of parotitis complicating appendicitis. The treatment is prophylactic and surgical. The former] consists in frequently cleansing the teeth, washing out the mouth with antiseptics, and removing the foul deposit from the tongue. Surgical treatment consists in the application of heat or cold to check the inflammation and in incision when pus is present. Rogers, Peck,[10] and others record cases of intestinal obstruction following operation for appendicitis. Fischl[11] wishes to emphasise the fact that there is undoubtedly such a disease as typhlitis, quite apart from any inflammation of the appendix. He gives an account of five cases, in all of which at some previous time the appendix had been removed. These patients all had symptoms which would assuredly have been diagnosed as appendicitis if their previous history had not been known. These cases all cleared up without operation under simple treatment, and the author thinks that the symptoms are referable to an inflammation of the cæcum. Bottentuit[12] also calls attention to some forms of pseudo-appendicitis which he calls entero-typhlocolitis and intestinal lithiasis. We have to deal for the most part with crises which simulate appendicitis, crises which may be called pseudo-appendicitis in contra-distinction to infective appendicitis. Some writers, however, say that muco-membranous, entero-typhlitis leads to appendicitis, and this may be true in some six or seven per cent. of cases. In intestinal lithiasis we almost always find in the vessel, along with sand, slimy and mucous substance and false membrane. Intestinal lithiasis nearly always coincides with entero-typhlocolitis, although some cases have been noted in which lithiasis existed alone. These salts are composed of phosphates and carbonate of lime and also of ammonio-magnesian phosphates. They also contained some organic matter. The presence of sand often sets up painful attacks which are mistaken for appendicitis. Cases of entero-typhlocolitis are usually easy to diagnose; the expulsion of slimy or mucous matter or false membrane, constipation or alternating constipation and diarrhœa, or diarrhœa alone, or pains in the abdomen, enable us to recognise the disease. Diagnosis is difficult only when there are paroxysmal crises. These crises may arise suddenly: errors of diet or a strong purgative, or fatigue, or any kind of excess, or even psychical causes may produce them. This pain is usually more severe on the right side over the hepatic flexure, and on the left side over the splenic flexure. Next in frequency to these are painful points in the iliac fossa on the right or left side, near the cæcum or sigmoid flexure. The patient is the subject of intestinal troubles, constipation, diarrhœa, especially under the influence of cold or departure from regular diet. In appendicitis the maximum of pain is in the cæcum, very near the appendix, but in entero typhlocolitis the pain is not definitely localised; it radiates over the path of the colon, principally on a level with the hepatic flexure and splenic flexure. Interesting cases are recorded by Lediard and Sedgwick[13] and Godwin.[14]

[1] Brit. Med. Jour., Oct. 8, 1904, p. 873. [2] Pract., June 1904, p. 829. [3] Med. Rec., June 18, 1904, p. 1025. [4] Edin. Med. Chi. Jour., March 1904, p. 200. [5] Clin. Jour., April 13, 1904, p. 413. [6] Dublin Jour. of Med. Sci., Aug. 1904, p. 153. [7] Med. Rec., July 2, 1904, p. 14. [8] Ibid., Aug 13, 1904, p. 266. [9] Lancet, July 30, 1904, p. 312. [10] Annals of Surgery, July 1904, p. 122, [11] Med. Chron., May 1904, p. 101. [12] Lancet, April 23, 1904, p. 1113. [13] Ibid, Sept. 10, 1904, p. 761. [14] Oct. 1, 1904, p. 951.

